# *Escherichia coli* O80 in Healthy Cattle: Absence of Shigatoxigenic and Enteropathogenic *E. coli* O80:H2 and (Phylo) Genomics of Non-Clonal Complex 165 *E. coli* O80

**DOI:** 10.3390/microorganisms11020230

**Published:** 2023-01-17

**Authors:** Rie Ikeda, Keiji Nakamura, Marc Saulmont, Audrey Habets, Jean-Noël Duprez, Nicolas Korsak, Tetsuya Hayashi, Damien Thiry, Jacques G. Mainil

**Affiliations:** 1 Bacteriology, Department of Infectious Diseases, Faculty of Veterinary Medicine, Centre for Fundamental and Applied Research for Animals and Health (FARAH), University of Liège, B-4000 Liège, Belgium; 2 Department of Bacteriology, Faculty of Medical Science, Kyushu University, Fukuoka 812-8582, Japan; 3 Association Régionale de Santé et d’Identification Animale (ARSIA), B-5590 Ciney, Belgium; 4 Food Inspection, Department of Food Science, Faculty of Veterinary Medicine, Centre for Fundamental and Applied Research for Animals and Health (FARAH), University of Liège, B-4000 Liège, Belgium

**Keywords:** healthy cattle, Shigatoxigenic *Escherichia coli*, enteropathogenic *Escherichia coli*, O80:H2, O80:H6, O80:H45, CC165, phylogenomics

## Abstract

The origin of human and calf infections by Shigatoxigenic (STEC) and enteropathogenic (EPEC) *Escherichia coli* O80:H2 is still unknown. The aim of this study was to identify *E. coli* O80 in healthy cattle with an emphasis on melibiose non-fermenting *E. coli* O80:H2. Faecal materials collected from 149 bulls at 1 slaughterhouse and 194 cows on 9 farms were tested with O80 antigen-encoding gene PCR after overnight growth in enrichment broths. The 53 O80 PCR-positive broths were streaked on different (semi-)selective agar plates. Five *E. coli* colonies from 3 bulls and 11 from 2 cows tested positive with the O80 PCR, but no melibiose non-fermenting *E. coli* was isolated. However, these 16 *E. coli* O80 were negative with PCR targeting the *fliC_H2_*, *eae*, *stx1*, *stx2* and *hlyF* genes and were identified by WGS to serotypes and sequence types O80:H6/ST8619 and O80:H45/ST4175. They were phylogenetically related to *E. coli* O80:H6 and O80:H45 isolated from different animal species in different countries, respectively, but neither to STEC and EPEC O80:H2/ST301, nor to other serotypes of the clonal complex 165. As a conclusion, healthy adult cattle were not identified as a source of contamination of humans and calves by STEC or EPEC O80:H2.

## 1. Introduction

Enterohemorrhagic *Escherichia coli* (EHEC) are a hybrid pathotype producing the Shiga toxins (Stx) of Shigatoxigenic *E. coli* (STEC) and the attaching–effacing (A/E) lesion of enteropathogenic *E. coli* (EPEC) [1]. Since the EHEC nomenclature is considered obsolete by EFSA [2], they will be named “Attaching-Effacing STEC” (AE-STEC) [3] in this manuscript.

The most frequent and pathogenic AE-STEC in humans belong to the following 6 serotypes: O26:H11, O103:H2, O111:H-, O121:H19, O145:H- and O157:H7 [1,4]. Nevertheless, other serotypes can emerge from time to time, either causing dramatic short-lived outbreaks, such as STEC O104:H4 in 2011 [5], or establishing themselves for longer periods, such as AE-STEC O80:H2 in France since ca. 2010 [6,7]. AE-STEC O80:H2 have also been reported in neighbouring countries (Belgium, Switzerland and the Netherlands), although not at the same frequency as in France [8,9,10]. Today, they represent the second- or third-leading cause of haemolytic uremic syndrome (HUS) in Europe [11,12]. In addition to haemorrhagic colitis and HUS, AE-STEC O80:H2 are responsible for systemic infections. Indeed, they harbour a pS88-like ColV plasmid carrying genes encoding virulence properties of extra-intestinal *E. coli* [6,13,14,15,16]. AE-STEC and EPEC O80:H2 belong to the sequence type (ST) 301 that is member of the clonal complex (CC) 165 along with other *E. coli* O80 and non-O80 serotypes, and different STs [17,18].

AE-STEC and EPEC O80:H2 have also been frequently identified in young diarrheic and, more rarely, septicemic calves in Belgium since 2009 [19,20]. They are highly related to human AE-STEC O80:H2 phylogenetically and by their virulotypes, including the presence of a pS88-related plasmid [8,19].

Ruminants, especially cattle, are considered the most frequent source of human infection by the classical AE-STEC serotypes via foodstuffs contaminated by their faecal materials, since they can be asymptomatic carriers in their intestines [1]. However, the European Food Safety Agency reports no detection of AE-STEC O80:H2 in food in 2019 and 2020 [11,12], although they have been sporadically isolated from healthy cattle and dairy products in Spain and France, but not as yet in Belgium, in the past [6,13,17,21,22]. One possible reason is that AE-STEC O80:H2 were present under the detection limits of the different methodologies applied during those surveys. The rate of isolation could be increased using the recently described melibiose-MacConkey agar, since no human AE-STEC O80:H2 ferment melibiose, in contrast to most other *E. coli*. This is due to the deletion of the melibiose operon (*mel*) associated with the insertion of a 70 bp long DNA fragment (*70mel*) [23].

The purpose of the present study was, therefore, to (i) isolate *E. coli* O80 from healthy bulls at the slaughterhouse and healthy cows in farms, with emphasis on AE-STEC and EPEC O80:H2; (ii) identify the newly isolated *E. coli* O80 by PCR; and (iii) understand their phylogenomic relationships within the *E. coli* species after whole genome sequencing (WGS).

## 2. Materials and Methods

### 2.1. Identification of O80 PCR-Positive Faecal Samples

In November and December 2020, 149 slaughterhouse faecal samples were collected from young bulls during 3 visits to 1 slaughterhouse in the province of Liège, Belgium. The bulls originated from 43 different herds in the provinces of Liège, Limburg, Luxemburg and Namur, Belgium. Between October 2021 and February 2022, 194 faecal samples were collected from the rectum of healthy cows in late pregnancy, or maximum 1 month after calving, on 9 farms located in the provinces of Liège and Luxemburg, Belgium. One gram of each faecal sample was added to 9 mL of lauryl sulphate broth (VWR Chemicals, Leuven, Belgium) and incubated overnight at 37 °C with shaking, for enterobacterial enrichment.

After centrifugation of 2 mL of each enrichment broth for 2 min at 13,000 RPM, the bacterial pellets were suspended in 100 µL of DNase free water (VWR Life Science, Leuven, Belgium) and total DNA was extracted by boiling for 10 min. In parallel, 2 mL of the enrichment broths was transferred into 2 CRYO tubes (Greiner Bio-One, Frickhausen, Germany) with 2 mL of sterile 80% glycerol and stored at −20 °C and −80 °C, respectively. The DNA samples obtained were subjected to PCR targeting the *wzy* gene in the O80 antigen-encoding gene cluster (referred to as O80 PCR; Table 1) using the FASTGENE2x Optima Hotstart kit (Nippon Genetics, Filter service, Eupen, Belgium). The amplification condition employed was as follows [24]: initial denaturation at 94 °C for 1 min, 30 cycles of annealing at 58 °C for 30 s, extension at 72 °C for 1 min, and denaturation at 94 °C for 30 s, and final extension at 72 °C for 2 min. The 285 bp-long amplicons were detected by electrophoresis in 1.5% agarose gel (VWR Life Science, Leuven, Belgium) in TAE buffer (Bio-Rad, Temse, Belgium) after staining with Midori Green (Nippon Genetics Europe, Düren, Germany).

### 2.2. Identification of O80 PCR-Positive E. coli

O80 PCR-positive enrichment broths were streaked on 5 (semi-)selective agar plates: Chromocult Coliform ES agar, Chromocult Coliform ES agar complemented with 2.5 mg/mL of potassium tellurite (TeK) and Chromagar STEC agar (CHROMagar, Paris, France) for both slaughterhouse and farm samples, and either Rapid *E. coli* 2/agar (Bio-Rad, Temse, Belgium) for slaughterhouse samples or MacConkey agar (VWR Chemicals, Leuven, Belgium) for farm samples. According to the manufacturers, Chromocult Coliform ES, Rapid *E. coli* 2/and MacConkey are selective for enterobacteria and coliforms in general, while TeK Chromocult Coliform ES and Chromagar STEC are selective for Te^++^-resistant coliforms, including a majority of STEC and EPEC.

All samples were also streaked on EnteroHemolysin (EHly) blood agar plates (Oxoid Deutschland, Wesel, Germany) to detect the production of enterohemolysin. After overnight incubation at 37 °C, up to 5 *E. coli*-like colonies were randomly picked up from the (semi-)selective agar plates. From the EHly blood agar plates, up to 10 colonies were picked up: 5 enterohemolyin-non-producing and 5 enterohemolysin-producing (if detected) colonies. In parallel, the O80 PCR-positive enrichment broths from farms were also streaked on melibiose-MacConkey agar plates. Melibiose-non-fermenting colonies (if detected) were picked up and subjected to species identification by API20E^®^ (BioMérieux, Craponne, France), following the manufacturer’s instructions.

O80 PCR was performed on all picked-up colonies as described above, using the DNA samples obtained from 2 mL of an overnight culture in Luria–Bertani (LB) broth (VWR Chemicals, Leuven, Belgium) at 37 °C with shaking.

### 2.3. PCR Typing

O80 PCR-positive *E. coli* isolates were further tested by PCR using the FASTGENE2x Optima Hotstart kit (Nippon Genetics, Filter service, Eupen, Belgium) to detect the *fliC_H2_* gene encoding the H2 antigen and the following virulence-associated genes: *eae* encoding the intimin adhesin involved in the A/E lesion, *stx1* and *stx2* encoding Stx1 and Stx2a to Stx2d toxins, and pS88-located *hlyF* encoding the avian haemolysin (Table 1). The amplification condition employed was as follows: initial denaturation at 95 °C for 15 min, 30 cycles of annealing at 55 °C for 90 s, extension at 72 °C for 90 s, denaturation at 94 °C for 30 s and final extension at 72 °C for 10 min [16,24,25]. The amplicons were detected by agarose gel electrophoresis, as described above.

### 2.4. Genome Analysis

The bovine O80 PCR-positive *E. coli* isolated during the abovementioned screening and 1 O80 PCR-positive isolate obtained from duck faecal material in 2009 were genome sequenced. Genomic DNA was purified from bacterial cells grown overnight in LB at 37 °C using NucleoSpin^®^ Microbial DNA (Macherey-Nagel, Düren, Germany). Libraries for Illumina sequencing were prepared using NEBNext UltraII FS DNA Library Prep Kit for Illumina (NEW ENGLAND BioLabs, Tokyo, Japan) and sequenced on the Illumina Miseq platform (Illumina) to generate 300 bp paired-end reads. Assembly of the Illumina sequence reads was performed using the SPAdes (v3.13.0) assembler [26]. Sequencing statistics of each isolate are shown in Table S1. Raw read sequences obtained in this study were deposited to GenBank/EMBL/DDBJ under the BioProject PRJNA906740.

Gene annotation and in silico H antigen-genotyping were conducted by using DFAST [27] and SeroTypeFinder 2.0 [28], respectively. The ST was determined by MLST 2.0 based on Achtman’s scheme of multi-locus sequence typing (MLST) [29]. Plasmid replicons were identified by using PlasmidFinder v2.1.6 [30]. Virulence-associated and antimicrobial resistance (AMR) genes were searched by VirulenceFinder v2.0.4 and ResFinder v.4.1.11, respectively [31,32]. pS88 plasmid-located genes were detected by BLASTN search of each genome in the DNA sequence dataset for the pS88-located genes associated with bacteriocin production and immunity (*cia*, *imm*, *cvaABC* and *cvi*), iron acquisition (*iucABCD*, *iutA*, *shiF* and *sitABCD*) and virulence (*iss*, *etsABC*, *ompT* and *hlyF*) [16] (Table S2). All searches were performed at a threshold of >90% identity and >60% coverage.

### 2.5. Database Search for Genomes of E. coli O80 Identified Serotypes

The NCBI and EnteroBase databases (final access: 4 October 2022) were searched for the genome sequences of *E. coli* belonging to the O80 serotypes identified during the abovementioned survey (Table 2). Their Illumina reads were downloaded and assembled using SPAdes as described above (Table S1). Using assembled genomes, ST determination and analyses of plasmid replicons, virulence-associated genes, AMR genes and pS88-encoded genes were performed as described above.

### 2.6. Phylogenomics

To understand the phylogenetic positions of the *E. coli* O80 identified in healthy cattle, closed chromosome sequences of 104 *E. coli* strains representing each of 104 serotypes were selected and downloaded from the NCBI database and annotated by DFAST (Tables S1 and S3). The chromosome sequence of *Escherichia* cryptic clade I strain TW10509 (No. AEKA00000000) was also downloaded and annotated to be used as an outgroup. The core genes (*n* = 2560) of all those *E. coli* were identified, and their concatenated sequence alignments were generated by Roary [33]. Based on the 97,551 SNP sites extracted from the alignment using SNP-sites [34], a maximum likelihood (ML) tree was constructed using RAxML [35]. Strains were deduplicated if the core sequences were identical. The phylogroup of each strain was determined by EzClermont [36] and the ML tree was displayed using iTOL [37].

## 3. Results

### 3.1. Identification of E. coli O80 in Faecal Samples from Slaughterhouse

**Figure 1:**
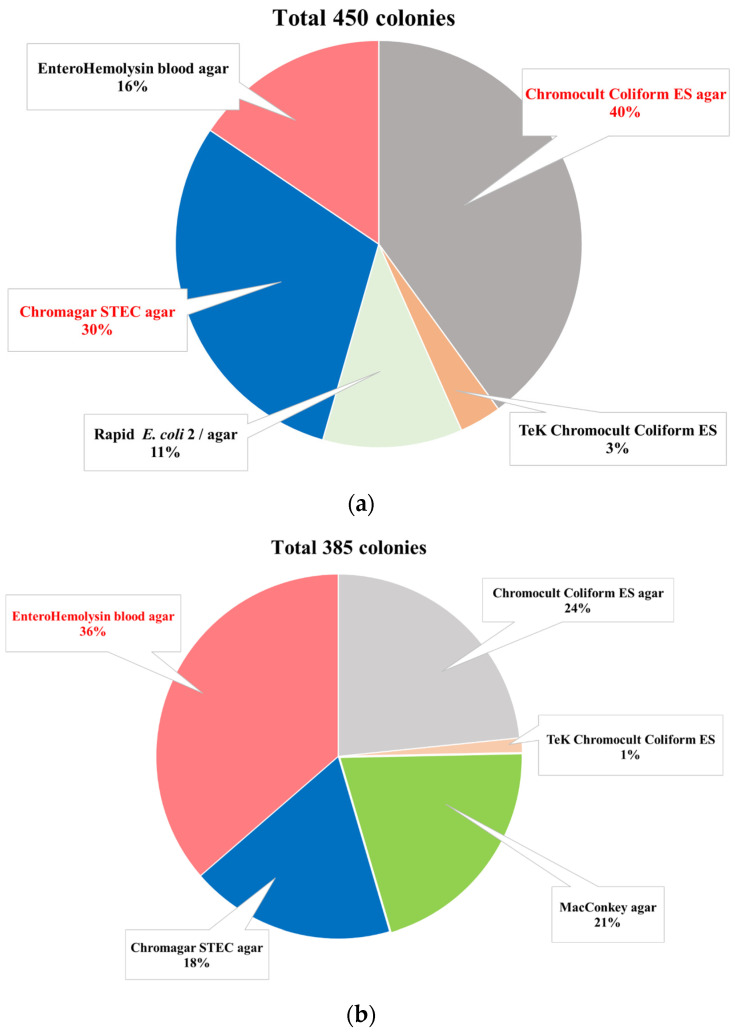
Proportions of the collected colonies from the different agar plates inoculated with the 35 O80 PCR-positive enrichment broths from slaughterhouse faecal samples (**a**) and with the 18 O80 PCR-positive enrichment broths from farm faecal samples (**b**).

After overnight enrichment growth in lauryl sulphate broths, 35 out of the 149 faecal samples (23%) from young bulls at 1 slaughterhouse were positive with the O80 PCR. Using the non-specific methodology, 450 colonies were picked up, with the majority from Chromocult Coliform ES (40%) and Chromagar STEC (30%) agar plates (Figure 1a). After performing the O80 PCR twice, 5 isolates (1%) from 3 faecal samples (2%) were confirmed as *E. coli* O80. Three of the 5 isolates were isolated from 2 bulls on the Chromocult Coliform ES agar and the remaining 2 from another bull on EHly agar.

### 3.2. Identification of E. coli O80 in Faecal Samples from Farms

After overnight enrichment growth in Lauryl Sulfate broths, 18 out of the 194 faecal samples (9%) from cows in 5 out of the 9 farms (55%) were positive with the O80-antigen PCR. Using the non-specific methodology, 385 colonies were collected, with the majority from EHly (36%) and Chromocult Coliform ES (24%) agar plates (Figure 1b). After performing the O80 PCR twice, 11 isolates (3%) from 2 cow faecal samples (1%) from 2 different farms (22%) were confirmed as *E. coli* O80. Ten of the 11 isolates from farm samples were isolated from 1 cow on Chromocult Coliform ES (5 isolates) and MacConkey (5 isolates) agar, and the remaining 1 from a second cow in another farm on EHly agar.

The 18 positive enrichment broths of faecal samples from cows in farms were also streaked on melibiose-MacConkey agar plates. Although melibiose non-fermenting colonies could be isolated after overnight growth, none of them was identified to *E. coli*.

### 3.3. Genomic Identification and Characterization

To further identify the O80 PCR-positive isolates to AE-STEC or EPEC O80:H2, PCR and WGS analysis were performed to detect the presence of the *fliC_H2_*, *eae*, *stx1*, *stx2* and *hlyF* genes. However, none of these 5 genes was detected in any of the 16 O80 PCR-positive isolates.

In silico analysis of the genome sequences of these 16 O80 PCR-positive isolates revealed that their H-serotypes were H6 (*n* = 10) or H45 (*n* = 6) (Table 2). All 10 *E. coli* O80:H6 were obtained from the same cow in the screening of farm samples. Of the 6 *E. coli* O80:H45, 1 was obtained from the second cow in the screening of farm samples and 5 were obtained from the 3 bulls in the screening of slaughterhouse samples. The O80:H6 and O80:H45 isolates belonged to ST8619 and ST4175, respectively (Table 2).

**Table 2:** *E. coli* O80:H6/ST8619 and O80:H45/ST4175 analysed in this study.

O:H/ST Genotype (Nr Isolates)	Isolation			BioSample No. (Bioproject PRJNA906740)	Data Source
	Source (Nr Isolates)	Country	Year		
O80:H6/	Cows (10)	Belgium	2022	SAMN32092024–SAMN32092033	This study
ST8619 (18) O80:H45/ ST4175 (15)	Turkeys (8) ^1^ Cow (1) Bulls (5) Duck (1) Cow (1) ^2^ Cow (1) Cow (1) ^2^ Unknown (1) Cattle (1) Pig (1) Pig farm soil (1) Cow manure (1)	USA Belgium Belgium Belgium Canada France Germany Poland UK UK UK USA	2018–2021 2022 2020 2009 2007 2010 2004 2016 2017 2015 2017 2020	SAMN11372876, SAMN12913176, SAMN1299068, SAMN18586312, SAMN20862110, SAMN23100074, SAMN25980720, SAMN26027222 SAMN32092034 SAMN32092019–SAMN32092023 SAMN32092035 SAMN14379539 SAMEA5619080 SAMEA5619042 ESC_TA7527AA ^3^ ESC_BB1134AA ^3^ SAMEA4645274 SAMN15488558 SAMN17058957	NCBI This study This study This study NCBI NCBI NCBI EnteroBase EnteroBase NCBI NCBI NCBI

^1^ One isolate belonged to ST12217, which is a single locus variant of ST8619 (Table S2). ^2^ These 2 isolates belonged to ST1301, which is a single locus variant of ST4175. ^3^ IDs in EnteroBase.

The core gene sequence analysis also revealed that the 10 *E. coli* O80:H6 obtained from the same cow in 1 farm were identical, as were the 4 *E. coli* O80:H45 obtained from 2 bulls at the slaughterhouse. Therefore, only 1 *E. coli* O80:H6 from this cow and 3 *E. coli* O80:H45 from 2 bulls and 1 cow were included in the phylogenomic and other genome sequence-based analyses.

### 3.4. Genetic Features and Phylogenomics of the E. coli O80:H6 and O80:H45

The genome sequences of these 4 bovine Belgian *E. coli* O80:H6 and O80:H45 were compared with the genome sequences of 8 O80:H6 and 8 O80:H45 strains obtained from the NCBI and EnteroBase databases, and of 1 Belgian *E. coli* O80:H45 previously isolated from duck faecal material (Table 2). The position of these 2 *E. coli* serotypes in the entire *E. coli* phylogeny was compared to the position of *E. coli* O80:H2.

While the 9 *E. coli* O80:H6 were either Belgian bovine (*n* = 1) or US turkey (*n* = 8) isolates, the *E. coli* O80:H45 (*n* = 12) were isolated from cattle, duck, pig and the environment, in different countries (Belgium, Canada, France, Poland, UK and USA). Like the Belgian bovine isolates, all but 1 US turkey *E. coli* O80:H6 belonged to ST8619, and the remaining 1 belonged to ST12217, a single locus variant (SLV) of ST8619 (Table 2 and Table S2). Similarly, 7 of the additional 9 *E. coli* O80:H45, including the Belgian duck isolate, belonged to ST4175, like the 3 Belgian bovine isolates, and the remaining 2 belonged to an SLV of ST4175, ST1301 (Table 2 and Table S2).

**Figure 2:**
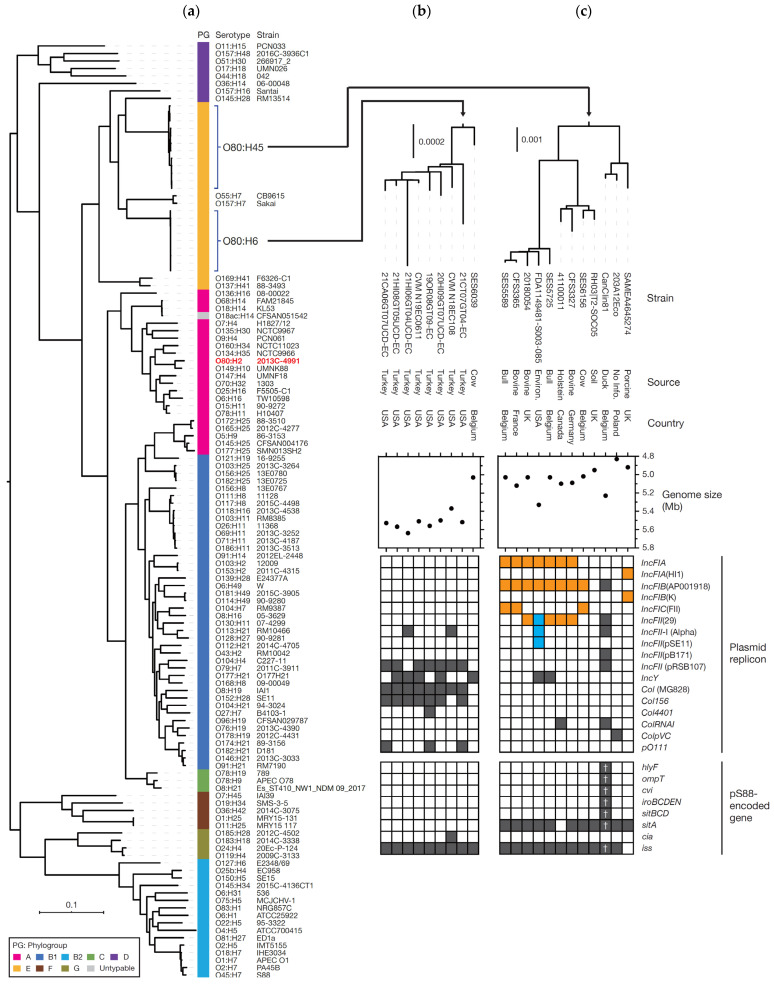
The phylogenetic positions and genetic features of the analysed *E. coli* O80:H6 and O80:H45, including genome sizes and distribution of plasmid replicons and pS88-located genes. In the left panel (**a**), a core gene-based ML tree of 104 chromosome-closed *E. coli* strains with an *Escherichia* cryptic clade I strain TW10509 (No. AEKA00000000) as an outgroup is shown along with names and serotypes of each strain. The tree was constructed based on the 97,551 SNPs identified in 2560 core genes. Phylogroups of each strain are also indicated. The right panels show the fine phylogenetic relationships of the 9 O80:H6 (**b**) or of the 12 O80:H45 (**c**) strains along with strain information. The genome sizes and the results of plasmid replicon and pS88-encoded gene search are also shown. The presence/absence of each replicon and gene is indicated by a filled/open box. The replicons found in the same contig in each strain are indicated by orange and cyan boxes. Genes nearly identical to those of pS88 (threshold: >99% identity and 100% coverage) are shown by daggers. Bar: the mean number of nucleotide substitutions per site.

The core gene-based phylogenetic analysis of the *E. coli* O80:H6 and O80:H45 and of the 104 *E. coli* whose chromosome sequences were downloaded from the NCBI database (Table S3) revealed that all 21 *E. coli* O80:H6 and O80:H45 belonged to phylogroup E and formed 2 single clusters, far distantly related to the AE-STEC and EPEC O80:H2 and to the clonal complex CC165 (Figure 2a).

#### 3.4.1. Genetic Features of *E. coli* O80:H6 Isolates

Phylogenetically, the Belgian bovine isolate (SES6039) formed a distinct branch from the 8 US turkey isolates (Figure 2b). Moreover, its genome size was significantly smaller (5033 kb vs. 5367 kb–5566 kb) and genome analysis by PlasmidFinder revealed the presence of only 1 plasmid replicon (*IncY*) in the Belgian bovine isolate, whereas the US isolates contained 3 to 5 replicons.

A search of virulence-associated genes by VirulenceFinder (Figure S1) revealed the presence of a range of potentially virulence-related genes in all 9 *E. coli* O80:H6. In addition, the *cib* gene encoding the Colicin Ib and the genes for the biosynthesis of pyelonephritis-associated pili (PAP) were detected in 1 and 2 US isolates, respectively. Conversely, no horizontally acquired AMR gene was detected in the Belgian bovine isolate by ResFinder (Figure S1), while 1 to 5 AMR genes were detected in the US turkey isolates.

#### 3.4.2. Genetic Features of *E. coli* O80:H45 Isolates

The 12 O80:H45 isolates (3 Belgian bovine, 1 Belgian duck and 8 other isolates) formed 3 distinct sub-clusters (Figure 2c) that are highly heterogeneous in terms of regions and sources of isolation. Of the 3 Belgian bovine isolates, 2 belonged to the same sub-cluster, although they are not closely related, and the third one to another sub-cluster. The duck isolate belongs to the third sub-cluster. Notable variation in genome size was also observed, even in the same sub-cluster (ranging from 4832 kb to 5331 kb) (Figure 2c). Plasmid replicon search revealed that the isolates containing the largest and second largest genomes (FDA1149481-S003-085S isolated in the US and the Belgian duck isolate, respectively) contained more replicons (7 and 6 replicons, respectively) than the other isolates (Figure 2c). Contigs carrying multiple replicons were present in 9 isolates (Figure 2c). In 8 of them, including the 3 bovine, but not the duck Belgian isolates, similar sets of 2 or 3 of the *IncFIA*, *IncFIB*(AP001918), *IncFIC*(FII) and/or *IncFII*(29) replicons were detected and, in 1 of these 8 isolates (FDA1149481-S003-085S with the largest genome), an additional contig containing 3 replicons was detected.

Search of virulence-related genes by VirulenceFinder (Figure S1) revealed that, in addition to a set of potentially virulence-related genes in all 12 *E. coli* O80:H45, some isolates contained additional virulence-related genes: *afaAB* (regulator and chaperone for afimbrial adhesins) in a French isolate, *cdtB* (B subunit of Cytolethal distending toxin) in a Canadian isolate, and different pS88-located genes in addition to *sitA* and *iss* (*hlyF*, *iroBCDEN*, *cma*, *ompT*, *cvi*, *sitBCD* and *cvaC*) in the Belgian duck isolate (Figure 2c and Figure S1). In the search of horizontally acquired AMR genes by ResFinder, AMR genes were only detected in the UK porcine isolate, which contained 9 AMR genes (Figure S1) conferring resistance to 8 antibiotic families.

## 4. Discussion

Although AE-STEC and EPEC O80:H2/ST301 emerged in humans and in calves more than a decade ago, there is still a lack of knowledge about their epidemiology. In comparison with several other AE-STEC serotypes, cattle are highly suspected as the source of contamination, since they can be asymptomatic carriers in their intestines [1]. However, surveys to isolate AE-STEC or EPEC O80:H2 from healthy adult cattle or young calves have been so far unfruitful, with a very few sporadic exceptions [6,13,17,21,22].

Using the same non-selective methodology as previously [22], a majority of AE-STEC and EPEC are expected to grow on the TeK Chromocult Coliform ES and Chromagar STEC agar media at the opposite of the majority of non-STEC non-EPEC strains that are Te^++^-sensitive [38]. Surprisingly, however, very few colonies from the 53 O80 PCR-positive enrichment broths grow on the TeK Chromocult Coliform ES agar plates compared to Chromagar STEC plates (Figure 1). The authors have no explanation for these different results between the 2 agar media, results that were not observed at such a scale in the previous study [22].

A total of 16 *E. coli* O80 were isolated from 5 of the 53 O80 PCR-positive enrichment broths (9%), although many more colonies from farm (36%) and slaughterhouse (16%) samples produce an enterohemolysin on the EHly agar (Figure 1), like AE-STEC and EPEC O80:H2 [6,15,19,20]. However, these 16 *E. coli* O80 belong to serotypes O80:H6 and O80:H45, and to ST8619 and ST4175, or their SLVs (Table 2 and Table S2), respectively. The most probable reason for this negative result is that *E. coli* O80:H2 was present, if at all, under the detection limits of this methodology. Testing more colonies is one alternative to increase the probability of isolating AE-STEC and EPEC O80:H2 but would be time- and labour-consuming. Another alternative is the use of a specific agar medium.

Therefore, melibiose-MacConkey agar plates [23] were streaked with the O80 PCR-positive enrichment broths of the faecal samples from the cows in farms, but no melibiose non-fermenting *E. coli* could be isolated. The reasons for this recurring negative result can be: (i) the human stool samples tested by Bizot and collaborators were clinical samples, probably with high numbers of AE-STEC O80:H2, while the bovine faeces tested in this survey were sampled from healthy animals, most probably with much lower numbers of *E. coli* O80:H2, if any, and (ii) the melibiose-MacConkey agar tested with the human stool samples also contains piperacillin, which was not used during our survey. Identifying the antibiotic/heavy metal, including to Te^++^, resistance profiles of the bovine AE-STEC and EPEC O80:H2 sequenced to date [17,19,39] will help to design more selective enrichment broths and agar plates to increase the rate of successful isolation. The rate of isolation of *E. coli* O80 could also be increased by designing an O80 antigen-specific capture method, like for other highly pathogenic AE-STEC serotypes [40,41].

*E. coli* O80:non-H2 from humans and animals have already been reported and can belong to one of the numerous *E. coli* pathotypes [1,17,42,43]. Like AE-STEC and EPEC O80:H2, some of them (O80:H19 and O80:H26) belong to phylogroup A and CC165 [17,18]. Nevertheless, neither the bovine *E. coli* O80:H6 and O80:H45 isolated during this survey, nor the 16 *E. coli* O80:H6 and O80:H45 whose genome sequences were downloaded from NCBI and EnteroBase databases, nor the additional Belgian duck *E. coli* O80:H45, are closely related to CC165 and members of phylogroup A (Figure 2).

Within serotype O80:H6, the only Belgian bovine isolate is placed in a distinct branch, but the other O80:H6 isolates were all isolated from turkeys in the USA (Figure 2). Within serotype O80:H45, the 3 Belgian bovine isolates are present in 2 different sub-clusters, along with other European and American bovine-related isolates (Figure 2), while the duck isolate is located in a third sub-cluster. Regarding the differences of the genome size within either serotype, the presence of more plasmid replicons in the isolates with the larger genomes suggests that the differences in plasmid content are partly contributing to the differences in genome size (Figure 2). Moreover, the presence of similar sets of replicons in 7 of the *E. coli* O80:H45 suggests that similar plasmids may be distributed in these strains, even though these 7 isolates are not related geographically, nor by their origins, nor by the year of isolation (Figure 2; Table 2 and Table S2). Clearly, more isolates of both serotypes are needed to refine their phylogenomic analysis, including the determination of complete genome sequences, before discussing further these results and hypotheses.

Search of virulence-associated genes by VirulenceFinder (Figure S1) revealed that no *E. coli* O80:H6 or O80:H45 are either (AE-)STEC, or EPEC, or belong to any other classical *E. coli* pathotype in humans or animals, with the possible exception of the duck *E. coli* O80:H45. Indeed, with the exceptions of the *afa* and *cdtB* genes in 2 O80:H45 isolates, the majority of the potentially virulence-related genes detected in the other *E. coli* O80:H6 and O80:H45 are widely distributed in *E. coli*, including in the laboratory strain K-12. Therefore, their importance in their pathogenicity, if any of these *E. coli* O80:H6 and O80:H45 is unclear at this stage, although some of them (*sitA* and *iss*) are involved in the survival of ExPEC in blood stream and internal organs and are plasmid-located [16,43].

Conversely, the duck *E. coli* O80:H45 harbours several pS88-located genes, including the *hlyF* gene (Figure 2) which is a marker of the virulence plasmids of ExPEC, avian pathogenic *E. coli* (APEC), AE-STEC and EPEC O80:H2 [16,17,19,39,44]. A more detailed examination of the putative pS88-located genes detected in the *E. coli* O80:H6 and O80:H45 of this study (Figure 2 and Table S1) was therefore performed. The *sitA* gene detected in most *E. coli* O80:H45 (11/12) has 100% identity to the *sitA* gene of pS88 in the duck isolate vs. 97.9–98.0% identity in the other isolates. Similarly, the *iss* gene detected in all *E. coli* O80:H6 (9/9) and most *E. coli* O80:H45 (11/12) also possesses 100% identity to the pS88-located *iss* gene in the duck isolate vs. 90.4–95.9% identity in the other isolates. In addition, nearly half (13/27) of the pS88-located genes are present in the duck isolate, with >99% sequence identity and 100% coverage to the pS88-located genes, and not in the other isolates. Finally, this duck isolate contains 1 of the 2 replicons of pS88 (*IncFIB*(AP001918)). These different data strongly suggest that the duck isolate, in contrast to the other *E.coli* O80:H6 and O80:H45, including the Belgian bovine ones, harbour a pS88-like plasmid. pS88 plasmids have already been detected in APEC O80:H26 [17], but to the authors’ knowledge, this is the first description in *E. coli* O80 outside of the CC165. Nevertheless, this duck *E. coli* O80:H45 was isolated from the faecal material and its actual virulence potential is not known at this stage.

Regarding horizontally acquired AMR genes, searching by ResFinder was negative for the Belgian bovine *E. coli* O80:H6 and O80:H45 (Figure S1). A few AMR genes are present in the US turkey *E. coli* O80:H6, but only the porcine *E. coli* O80:H45 can be genetically defined as a multidrug-resistant strain, with 9 AMR genes conferring resistance to 8 classes of antimicrobials (Figure S1). These results do not, however, exclude the existence of other non-horizontally acquired antimicrobial resistance mechanisms [45].

## 5. Conclusions

As general and specific conclusions, neither AE-STEC nor EPEC O80:H2 were isolated during this survey, and healthy adult cattle were not identified as the source of contamination of calves and humans. Moreover, the bovine *E. coli* O80:H6 and O80:H45 isolated during this survey are neither AE-STEC nor EPEC and phylogenetically, are only distantly related to the AE-STEC and EPEC O80:H2 or to the other *E. coli* O80 serotypes of the clonal complex CC165. More surveys targeting other putative sources of contaminations, such as the environment and wildlife, should be performed using selective methodologies to identify the source of contamination of humans and calves by AE-STEC or EPEC O80:H2. Further experiments should also be conducted to refine the (phylo)genomics and to assess the virulence potential, if any, of the Belgian bovine *E. coli* O80:H6 and O80:H45, and of the Belgian duck *E. coli* O80:H45.

## Figures and Tables

**Table 1:** Target genes, primer sequences and amplified fragment lengths of the PCR.

PCR	Target Genes	Primer Sequences	Amplified Fragments	Reference
O80	*wzy_O80_*	Og80-F: 5′-TGGTGTTGATTCCACTAGCGT-3′ Og80-R: 5′-CGAGAGTACCTGGTTCCCAAA-3′	285 bp	[24]
H2	*fliC_H2_*	Hg2-F: 5′-TGATCCGACACTTCCTGATG-3 Hg2-R: 5′-CCGTCATCACCAATCAACGC-3′	228 bp	[25]
Intimin	*eae*	SK2-F: 5′-CCCGGATCCGTCTCGCCAGTATTCG-3′ SK1-R: 5′-CCCGAAATCGGCACAAGCATAAGC-3′	881 bp	[24]
Stx1	*stx1*	LP44-F: 5′-CACCAGACAATGTAACCGCTG-3′ LP43-R: 5′-CAGTTAATGTGGTGGCGAAGG-3′	348 bp	[24]
Stx2	*stx2a* to *stx2d*	LP31-F: 5′-GCGTCATCGTATACACAGGAGC-3′ LP30-R: 5′-ATCCTATTCCCGGGAGTTTACG-3′	584 bp	[24]
Avian hemolysin	*hlyF*	HlyF-F: 5′-GGCGATTTAGGCATTCCGATACTC-3′ HlyF-R: 5′-ACGGGGTCGCTAGTTAAGGAG-3′	599 bp	[16]

## Data Availability

Not applicable.

## Supplementary Materials

The following supporting information can be downloaded at: https://www.mdpi.com/article/10.3390/microorganisms11020230/s1; Figure S1: Virulence-associated and antimicrobial resistance (AMR) genes detected in *E. coli* O80:H6 and O80:H45; Table S1: *E. coli* O80:H45 and O80:H6 strains used in WGS analyses; Table S2: The pS88-located genes used for repertoire analysis as references; Table S3: *E. coli* strains used for the construction of a core gene-based phylogenetic tree.
