# Supplementary material for: Escherichia coli O80 in Healthy Cattle: Absence of Shigatoxigenic and Enteropathogenic E. coli O80:H2 and (Phylo) Genomics of Non-Clonal Complex 165 E. coli O80
Source: Microorganisms. 2023 Jan 17;11(2):230. doi: 10.3390/microorganisms11020230 (PMC9962692; doi:10.3390/microorganisms11020230)
Supplement: Supplementary file 1 [file microorganisms-11-00230-s001.zip › Figure S1.pdf]

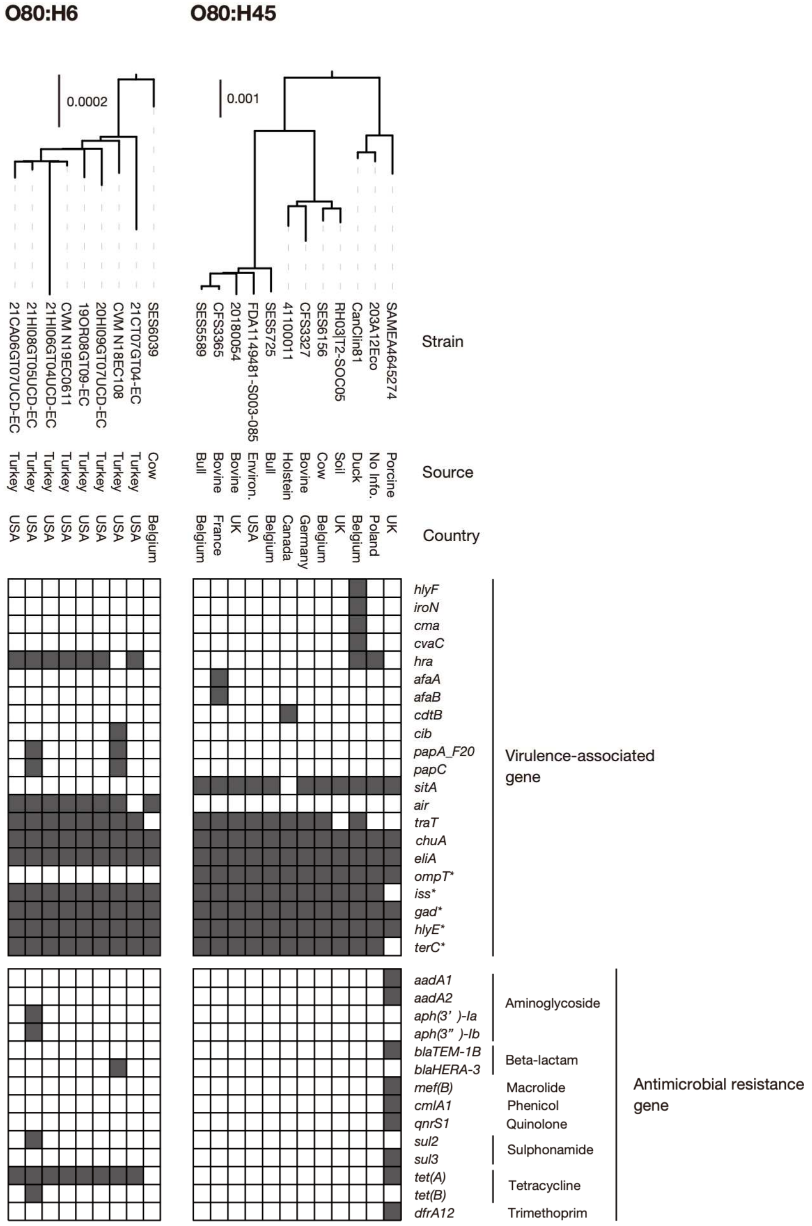

Figure S1. Virulence-associated and AMR genes found in O80:H6 and O80:H45 strains.

The results of gene search by using VirulenceFinder and ResFinder are mapped to the ML trees of O80:H6 and O80:H45 (the same trees as shown in Figure 2 in the main text). The presence/absence of each gene is indicated by a filled/open box. Genes found in *E. coli* strain K-12 (No. NC_000913) are indicated by asterisks. Bar, the mean number of nucleotide substitutions per site.
